# Progress in General Surgery

**Published:** 1900-12-22

**Authors:** 


### Progress in General Surgery.

**Fractures and Dislocations.—Upper Extremity.**—A very rare instance of fracture through the neck of the scapula is reported by Dr. J. Brown.[10] The arm was an inch longer than its fellow, and there was flattening over the deltoid with marked prominence of the acromion. The deformity disappeared on pulling the elbow upwards, while rotation of the arm elicited distinct crepitus. On allowing the arm to hang the deformity disappeared. Pressure over the coracoid caused pain with reappearance of the deformity, and this process could be moved independently of the scapula. Under either the parts were accurately adjusted, and the arm strapped to the side over a firm pad in the axilla. The arm was well supported under the elbow and across the opposite shoulder by strapping, flannel, and plaster bandages. Union was perfect. In reporting two cases of unreduced dislocation of the elbow Loison[11] says that no treatment short of partial resection can have any good result. Passive movement, massage, subcutaneous section of fibrous adhesions, and even arthrotomy usually fails, in consequence of the development of ossifying periarthritis and of osteophytes both on the bones and as detached bodies in the surrounding muscles. Like Ollier, he removes the lower end of the humerus, and leaves intact the upper end of the ulna with the olecranon, so as to preserve the attachments of the biceps and brachialis anticus. Dr. F. J. Cotton[12] has studied in detail the lesions found in dissected specimens of fracture of the lower end of the radius, using the skiagraph and experimental evidence only as verifications. He includes under the term of Colles's fracture all those fractures of the radius which occur within two inches of the wrist joint. All these fractures have certain characteristics which are, broadly speaking, constant—the proximity to the joint, the displacement and rotation of the lower fragment upwards, backwards, and outwards, the forward displacement of the ulna, &c. The variations from this type which occur bear no obvious relation to the variations in the detailed bone lesions, however, and a broad grouping is hardly possible. As regards separation of the lower radial epiphysis, he states that this lesion has been most carefully studied by Vogt, Bruns, De Paoli, Bennett, and lately in the wonderfully complete monograph on epiphyses by Poland. Clinically, the injury is far less common than Colles's fracture: but opportunities for study have been many, for a very large proportion of cases are compound, and many deaths from sepsis and many amputations swell the list of specimens. Moreover, the injury seems to be, in larger degree than Colles's fracture, a product of severe trauma, and is relatively often found in children dead from severe falls. In all there are sixty specimens of which accurate examinations are at hand. These include, according to Poland, fourteen compound cases. Forward displacement seems not very unusual. Poland cites five instances. Most interesting of these epiphysial separations are those without displacement, and the partial separations. Of the latter class D'Arcy Power described a dissected specimen, and since the study of those legions with the X-ray has attracted attention to them, many clinical cases are to be found. They give the signs of local damage and sometimes mobility; the X-ray shows almost nothing. The complete separations are usually the result of falls, and often (according to De Paoli in nineteen of thirty-five; according to Poland an even larger proportion, thirty-eight of forty) from falls on the palm. Falls on the palm in young patients with unconsolidated epiphyses, where the trauma is less, seem to lead either to fracture above the epiphysial line or to a simple springing and wrenching of the epiphysial line, evidenced by extreme well-localised tenderness, and by an appearance in the skiagraph of a slight widening of the line (not constantly present, and likely to be obvious on one side only). Dr. Jno. E. Prichard[13] relates his own case of the so-called "stave of thumb" or Bennett's fracture caused by a fall from a bicycle a year previously. He did not diagnose it at first on account of the swelling and the absence of crepitus, but the latter could be detected a fortnight after the accident. The thumb could not be opposed to the other digits; this inability remained for three months. The case was practically untreated, and now that union had taken place in a faulty position a characteristic deformity remained; that is, the proximal end of the shaft of the bone was drawn upwards and slightly forwards by its extensor tendon, and appeared as a swelling in the space between that tendon and the tendon of the extensor secundi internodii, obliterating the "snuff-box."

**Lower Extremity.**—An interesting case of separation of the epiphysial head of the femur is recorded by J. Jackson Clarke[14] in a girl aged fifteen years. The hip had been injured by a fall five months previously, and the lower extremity was in the position of marked coxa vara, with great loss of flexion and adduction. Mr. Poland, under whose care the patient was in the City Orthopædic

Hospital, removed the head of the bone and a good deal of callus by an anterior incision, and placed the end of the neck in the acetabulum. The ultimate result was in every way successful. The following case is recorded by Lucas-Championnière,[15] as an instance of the unreliableness of skiagraphy, and he believes that this method, unless carefully and conscientiously practised, is likely to injure the reputation of the practitioner. A patient with a perfectly sound and useful lower limb, after complete recovery from a contusion of the thigh, was led by a skiagraph to believe that he was the subject of an overlooked and ununited fracture of the femur. A shadow in the upper part of the bone cast by the ischium and a clear band below this were supposed to represent the serious nature of the injury. For fracture of the patella Dr. W. B. Hopkins [16] recommends a special extension apparatus, consisting of a series of adhesive straps applied to the thigh in an intersecting diagonal form, each strap terminating in a ring at the knee, one half of the series being attached at the outer, and the other half at the inner side. The ordinary extension cords connect the rings with the pulley-cord over the foot of the bed. It acts like the familiar Indian finger puzzle, which, slipping readily on the finger, tightens as traction is made on it. This traction exerts two distinct forces upon the thigh, viz., extension downwards and constriction. The former draws down the integument of the thigh and, by means of a bridle placed across it, the upper fragment of the patella. The latter, by exerting a continuous unremitting constriction, paralyses the muscles of the thigh, without causing the distal portion of the limb to swell at all from impeded circulation. Although the knee-joint is not controlled, the disabling effect on the flexors, as well as on the extensors of the thigh, prevents the patient flexing his leg. Passive movement of the leg is commenced from the first within 10 or 15 degrees, and is conducted without disturbing the fragment, by simply elevating the knee from the bed. The apparatus is usually kept on for seven weeks, but may require renewal during that time. It is an extremely comfortable one for the patient, and is usually applied on the fifth day, after the first inflammatory reaction has begun to subside. Eight pounds weight is generally sufficient, but more may be added. Complete motion of the knee-joint is ultimately restored. Dr. L. C. Hull[17] reports the following case of irreducible backward dislocation of the knee-joint. A lady was thrown from her horse, alighting, as she believes, on her feet, and then falling to the ground. When seen, seven hours later, in addition to a wound over the internal condyle of the femur, exposing the bone, the patella was missing from the intercondyloid notch, and the bones of the leg, notwithstanding the great swelling of the joint, were situated high up in the popliteal space. The leg was shortened, rotated laterally, very much swollen, and cold from the obstructed circulation: under chloroform, a prolonged attempt was made at reduction, without success. On the following day another unsuccessful attempt was made. On account of the infected wound and the surroundings of the patient no operative procedure was deemed advisable. But after the healing of this wound and of another which formed over the external condyle the leg was found to be shortened four inches, and had no support except the soft structures behind the knee, pressure against which caused pain and swelling. It being evident that the leg was useless, resection of the knee-joint was performed eight months after the accident. The patella was found surrounded by dense adhesions and perfectly fixed near the head of the fibula. The ligamentum patellæ and quadriceps femoris were intact; after removal of the articular surfaces of the femur and tibia it was necessary to divide both hamstring tendons before the bones could be brought into positions. They were then held in position by Wyeth's fixation drills. At the end of seven weeks the patient could bear her whole weight on the leg, the bones having firmly united. Two years after the accident she could walk without discomfort, and the leg was as strong as ever. A rare instance of traumatic dislocation forwards of the head of the fibula without fracture is reported by R. J. Pye-Smith.[18] A goods porter, aged 24, had his knee crushed between a heavy door and some iron plates. The limb presented a peculiar twisted appearance, due to slight eversion of the leg and foot and to prominence of the head of the fibula. There was no effusion into the knee, and its passive movements could be easily and perfectly effected. Active movements could be performed with pain. There was tenderness over the head of the fibula, but no mobility or crepitus. The head of the fibula was ½ inch nearer to the tubercle of the tibia in front than on the sound side, but ¾ inch further from it when measured round the posterior aspect. The peroneal nerve and biceps tendon were intact. Under ether an attempt at reduction of the dislocation was made by direct pressure backwards by the thumbs on the head of the fibula, but the bone was too firmly fixed. The foot was then forcibly inverted, and backward pressure made on the head of the fibula, and reduction effected with an audible and palpable thud. The appearance of the limb at once became normal, the appearance of slight genu vulgum entirely disappearing. There was no tendency to recurrence of dislocation, but a back splint was applied for a few days. Rapid recovery ensued, and the patient resumed his work two or three weeks later. R. J. Pye-Smith [19] also gives a skiagram and report of a case of dislocation of the foot backwards, in which he removed a large part of the astragalus, with a successful result. The injury occurred in a man, aged 34, three or four months previously, by his slipping on a stone and twisting his foot. Splints and a plaster case had been applied without any success. The skiagram showed, besides the backward displacement, fracture and displacement of a small portion of the articlar end of the tibia and a nodule of new bone behind it. A case of dislocation inwards of the foot, without fracture, is related by W. A. Payne,[20] in a man, aged 33, who on stepping on to a plank was thrown forcibly on to his left side. The sole of the foot was found to be parallel to the floor. The foot was pushed inwards and the internal malleolus rested on the middle of the trochlear surface of the astragalus. The external lateral ligament was ruptured; the internal one was not, and could be felt as a taut band running from the malleolus almost horizontally inwards to the displaced astragalus. On the outer side the malleolus had nearly penetrated the skin, and was felt beneath it as a sharp projection. After repeated efforts under chloroform the foot was forcibly replaced. Five weeks later the patient left the hospital with a useful and normal leg. F. A. Southam [21] reports a case of fracture of the os calcis, treated by wiring the fragments. A housekeeper, aged 62, fell off a step ladder, but did not know the position in which she did so. The whole of the posterior part of the foot was much ecclymosed and swollen, the normal depressions on either side of the tendo Achillis being quite obliterated; on the posterior surface of the heel a distinct hard transverse ridge could be felt beneath the skin but no crepitus. All power of flexion and extension of the foot was lost, and passive movement caused great pain. A skiagram clearly showed a detached wedge of bone, evidently torn off by the action of the calf muscles from the upper surface of the os calcis, leaving between the base of the wedge and the bone from which it had been detached an interval of an inch, explaining the absence of crepitus. Through a posterior incision a stout silver wire was passed through the upper fragment, close to the tendo Achillis, and through the posterior part of the body of the bone. The fragments were brought into accurate position, and the limb fixed on a straight outside splint in the extended position. Eleven days later the skiagram showed the silver wire *in situ*, and accurate apposition of the fragments. Passive movements was commenced on the fourteenth day, and a week later the foot placed in a plaster of Paris case. This was removed about six weeks later, when the patient could walk with a stick. The range of movement of the foot was almost perfect. W. A. Payne[22] alludes to a case of fracture of the fourth metatarsal bone in a man whose foot in the strongly extended position had, with the anterior part of the sole and toes, come in contact with the pavement. Swelling over the outer part of the foot, pain on pressing the fourth toe, crepitus, and false mobility at one point were the only signs discoverable.

[10] New York Med. Jour., June 23, 1900, p. 1013. [11] Ep. Brit. Med. Jour., July 14, 1900, and Rev. d'Ortho., Nov. 2, 1900. [12] An. of Surg., Aug. 1900, p. 194. [13] Lancet, June 23, 1900, p. 1533. [14] Lancet, Oct. 27, 1900. [15] Ep. Brit. Med. Jour., May 26, 1900. [16] An. of Surg., May, 1900, p. 633. [17] An. of Surg., Aug. 1900, p. 289. [18] Quar. Med. Jour., Aug. 1900, p. 408. [19] Ibid., p. 406. [20] New York Med. Rec., March, 1900, p. 361. [21] Med. Chron., July, 1900, p. 260. [22] New York Med. Rec., March, p. 362.

